# The Linear Noise Approximation for Spatially Dependent Biochemical Networks

**DOI:** 10.1007/s11538-018-0428-0

**Published:** 2018-04-11

**Authors:** Per Lötstedt

**Affiliations:** Division of Scientific Computing, Department of Information Technology, Uppsala University, SE-75105 Uppsala, Sweden

**Keywords:** Linear noise approximation, Spatially dependent, Fast algorithm, 60J60, 65C40, 92C45

## Abstract

An algorithm for computing the linear noise approximation (LNA) of the reaction–diffusion master equation (RDME) is developed and tested. The RDME is often used as a model for biochemical reaction networks. The LNA is derived for a general discretization of the spatial domain of the problem. If *M* is the number of chemical species in the network and *N* is the number of nodes in the discretization in space, then the computational work to determine approximations of the mean and the covariances of the probability distributions is proportional to $M^2N^2$ in a straightforward implementation. In our LNA algorithm, the work is proportional to $M^2N$. Since *N* usually is larger than *M*, this is a significant reduction. The accuracy of the approximation in the algorithm is estimated analytically and evaluated in numerical experiments.

## Introduction

Many biochemical networks are modeled by ordinary or partial differential equations at a macroscopic level of fidelity. Such continuous models may not be sufficiently accurate when the number of molecules involved in the chemical reactions is small. This is often the case in molecular cell biology (Elowitz et al. 2002; McAdams and Arkin 1997; Raj and van Oudenaarden 2008; Tsimring 2014). Chemical reactions are then best described as random events, and the discrete number of molecules is important when the copy numbers are low at a mesoscopic level of modeling. The macroscopic equation for the mean values is often satisfactory when the number of molecules is large. Analytical solutions to the governing macroscopic or mesoscopic equations can be obtained only for special systems. Computational methods are needed for quantitative information about the behavior of the systems.

The master equation (ME) or Kolmogorov forward equation is an equation for the time evolution of the probability density function (PDF) for the copy numbers of different species in systems with an intrinsic noise (Gardiner 2004; Gillespie 1992; van Kampen 2004). The systems are modeled as Markov processes with discrete states defined by the copy numbers of the chemical species in continuous time. The particular ME for spatially homogeneous, well-stirred problems in chemistry is the chemical master equation (CME) where reactions between two molecules occur with a propensity that depends on the copy numbers of the species. The ME is generalized in the reaction–diffusion master equation (RDME) to spatially heterogeneous chemical systems by introducing a discretization of the reaction volume into compartments or voxels (Gillespie et al. 2014; Gillespie and Seitaridou 2013). The state is then given by the copy numbers in each one of the voxels.

The computational work and the storage requirements to solve the RDME grows exponentially in the number of species and the number of voxels making the simulation of biochemical systems with the ME prohibitive except for very small systems. Analytical solutions are known only for limited classes of problems such as those with linear propensities. Instead, sample trajectories of well-stirred systems are generated by Gillespie’s stochastic simulation algorithm (SSA) (Gillespie 1976, 1977). The original algorithm has been improved in many ways, e.g., for efficiency (Cao et al. 2006; Gibson and Bruck 2000; Gillespie 2001) and for systems with slow and fast reactions (Cao et al. 2005; E et al. 2007). The Gillespie algorithm is generalized to problems with spatial variation due to diffusion in Elf and Ehrenberg (2004), Engblom et al. (2009), Isaacson and Peskin (2006), Lampoudi et al. (2009) and implemented in software (Drawert et al. 2012, 2016; Hattne et al. 2005). The computational effort may be quite large to simulate a system with many chemical species, many molecules, and many voxels, since many realizations of the process are required in a Monte Carlo method like SSA due to slow convergence to the mean and other moments of the distribution. An introduction and an overview of Markov models of chemical reactions are found in Goutsias and Jenkinson (2013). Recent reviews of computational methods at different levels of modeling are Engblom et al. (2017), Gillespie et al. (2013), Mahmutovic et al. (2012) and Sokolowski et al. (2017).

There are ways to approximate the solutions to the CME with deterministic equations. The *linear noise approximation* (LNA) is obtained from the CME by deriving the equations for the moments and then expanding the solution in a large parameter $\Omega$, representing the size of the chemical system (van Kampen 1976, 2004). The means and covariances in LNA are exact for chemical systems with at most first-order reactions where the propensities are constants or linear in the copy numbers. The first and second moments are exact also for other systems with a special structure (Grima 2015). Different modifications have been proposed to improve the accuracy of LNA, see, e.g., Ferm et al. (2008), Grima (2012). Some of the improvements are compared experimentally in examples in Schnoerr et al. (2015). The LNA and similar approximations are used to quickly study biochemical networks in, e.g., Elf and Ehrenberg (2003), Thomas et al. (2013), Ullah and Wolkenhauer (2009) and more recently as a surrogate model to infer parameters in biochemical models from data in Fearnhead et al. (2014), Fröhlich et al. (2016), Ruttor and Opper (2009), Stathopoulos and Girolami (2013). A review of LNA and related methods and their use for inference are found in Schnoerr et al. (2017).

An alternative to the LNA is the EMRE approximation in Grima (2010) extended to spatial problems in Smith et al. (2017). The covariances satisfy the same Lyapunov equation as we derive here. The spatial EMRE algorithm is applied to gene regulation in a cell and to reactions in a aggregation of cells in two space dimensions in Smith et al. (2017). More compartments than one are also found in Challenger et al. (2012). The equations of LNA with spatial variation are derived in Scott et al. (2010) and applied to the modeling of spatial patterns. The equation for the covariances is replaced by an equation for the factorial cumulant. Equations similar to the LNA for spatial problems are used in Butler and Goldenfeld (2009), Anna et al. (2010) to investigate oscillatory systems. Turing patterns are studied in Asllani et al. (2013), Biancalani et al. (2010), McKane et al. (2014), Woolley et al. (2011) with a spatially extended LNA.

Diffusive effects are important for the fidelity of models when the chemical reactions are localized in space in a cell and when the molecular transport is slow compared to the reactions. Some examples where the spatial effects are crucial are found in Fange and Elf (2006), Sturrock et al. (2013), Takahashi et al. (2010). The LNA is a level of modeling suitable for such systems, e.g., to infer parameters for the diffusion and the reactions from measurements, at least in the beginning of the iterative search process for the parameters, thanks to the relative simplicity of LNA.

In this paper, we develop a fast algorithm for computing approximations of the mean and the covariance of the PDF solving the RDME based on the LNA for spatial problems with reactions and diffusion. The equation for the expected values is a system of reaction–diffusion equations, and the equation for the covariances is a time-dependent Lyapunov equation with a source term localized in space. Let *M* be the number of chemical species and *N* the number of voxels. The structure of the covariance equations is utilized to compute an approximation of the covariance and to reduce the computational work and the memory requirements from being proportional to $M^2N^2$ in a straightforward implementation to $M^2N$ in our algorithm. Since *N* usually is larger than *M*, this is a substantial reduction. A bound on the deviation of the true covariance from our approximation is proved in a theorem. The accuracy of the covariance approximation is demonstrated in numerical examples in one, two, and three dimensions (1D, 2D, and 3D).

In the next section, the RDME is given and a splitting of the operator is introduced. The equations of the LNA for spatially heterogeneous chemical systems are derived for general shapes of the voxels in Sect. 3. A continuous approximation of the equation for the covariances is analyzed in Sect. 4. The algorithm is presented in Sect. 5 for computation of the mean and the covariance. Numerical results are found in Sect. 6. Finally, some conclusions are drawn.

The notation in the paper is as follows. The *i*th element of a vector $\mathbf v$ is denoted by $v_i$. The *j*th column of an array $\mathbf {x}$ with elements $x_{ij}$ is written as $\mathbf {x}_{\cdot j}$, and $\mathbf {x}_{i \cdot }$ is the *i*th row. The derivative of $v_i(\mathbf {x})$ with respect to $x_j$ is denoted by $v_{i,j}$. The time derivative $\partial p/\partial t$ of $p(\mathbf {x}, t)$ is written as $\partial _tp$, and $\dot{q}$ is a shorter notation for $\mathrm{d}q/\mathrm{d}t$. The Euclidean vector norm is denoted by $\Vert \mathbf v\Vert =\sqrt{\sum _{i}v_i^2}$ and the subordinate spectral norm for a matrix $\mathbf {A}$ is $\Vert \mathbf {A}\Vert$. The set of integer numbers is written as $\mathbb {Z}$, and $\mathbb {Z}_+$ denotes the nonnegative integer numbers. In the same manner, $\mathbb {R}$ denotes the real numbers and $\mathbb {R}_+$ is the nonnegative real numbers.

## The Master Equation

**Fig. 1:**
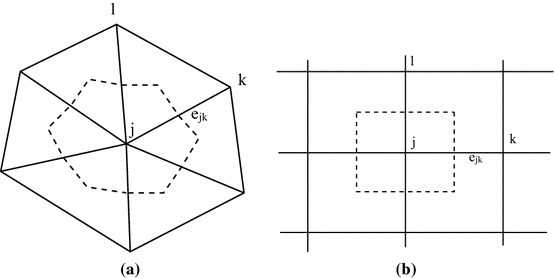
Meshes with edges (solid), nodes *j*, *k*,  and *l*, and voxel $\mathcal {V}_j$ (dashed). The nodes *j* and *k* are connected by edge $e_{jk}$. **a** An unstructured mesh, **b** a structured Cartesian mesh

Consider a biochemical system with *M* chemically active species. The system evolves on a line (1D), in an area (2D), or a volume (3D) $\mathcal {V}$ which is partitioned into *N* voxels (or compartments) $\mathcal {V}_j$ such that they cover $\mathcal {V}$, $\mathcal {V}=\bigcup _{j=1}^N \mathcal {V}_j$, and are non-overlapping, $\mathcal {V}_j\bigcap \mathcal {V}_k=\emptyset ,\; j\ne k$. The voxels are defined by a computational mesh constructed for numerical discretization of partial differential equations, see Fig. 1. The size of a voxel is $V_i=|\mathcal {V}_i|$, and the diagonal matrix $\mathbf V$ has the elements $V_i$ in the diagonal. Each voxel has a node in the center with coordinates $\mathbf {x}\in \mathbb R^d,\; d=1,2,3$ and the nodes are connected by edges. The molecular copy number of species *i* in voxel *j* is a random integer $Y_{ij}$. The state of the system is time dependent and is given by $\mathbf {y}(t)$ which is an array of nonnegative integers, $\mathbf {y}\in \mathbb Z_+^{M\times N}$. The state changes randomly with reactions between the species in a voxel and with diffusive jumps of the molecules between the voxels.

The CME is a Kolmogorov forward equation for the PDF $p(\mathbf {y}, t)$ for a system to be in the state $\mathbf {y}$ at time *t* (Gardiner 2004; van Kampen 2004). The state changes at discrete time points after a chemical reaction in a voxel. If $\tilde{\mathbf {y}}$ is the state before the reaction and $\mathbf {y}$ is the state immediately after the reaction *r*, then the change in state in $\mathcal {V}_j$ can be written as

1 $$\begin{aligned} \tilde{\mathbf {y}}_{\cdot j}\xrightarrow {w_r(\tilde{\mathbf {y}}_{\cdot j})} \mathbf {y}_{\cdot j},\quad \mathbf n_r =\mathbf {y}_{\cdot j}-\tilde{\mathbf {y}}_{\cdot j}. \end{aligned}$$

The reaction occurs with the propensity $w_r$, i.e., with probability $w_r\Delta t$ in a short time interval $\Delta t$. The state change vector $\mathbf n_r\in \mathbb Z^M$ tells how the state is updated after a reaction. Most entries of $\mathbf n_r$ are zero and $n_{ri}\ne 0$ only for those species involved in the reaction. In a system with *R* different reactions, the CME for $p(\mathbf {y}, t)$ is

2 $$\begin{aligned} \partial _tp(\mathbf {y},t)= & {} \displaystyle {\sum _{j=1}^N\sum _{r=1}^{R} w_r(\mathbf {y}_{\cdot j}- \mathbf n_r, t)p(\mathbf {y}_{\cdot 1}, \mathbf {y}_{\cdot 2},\ldots , \mathbf {y}_{\cdot j}- \mathbf n_r,\ldots , \mathbf {y}_{\cdot N}, t)} \nonumber \\&\displaystyle {-\sum _{j=1}^N\sum _{r=1}^{R} w_r(\mathbf {y}_{\cdot j}, t)p(\mathbf {y}, t)} \equiv \mathcal {M}p(\mathbf {y}, t), \end{aligned}$$

defining the master operator $\mathcal {M}$.

Diffusion of the molecules is modeled as jumps between voxels with a common boundary. Suppose that $\mathcal {V}_j$ and $\mathcal {V}_k$ share a point in 1D, an edge in 2D, or a facet in 3D. Then, a molecule of species *i* in $\mathcal {V}_j$ jumps to $\mathcal {V}_k$ with propensity $q_{jk}\tilde{y}_{ij}$

3 $$\begin{aligned} \tilde{y}_{ij}\xrightarrow {q_{jk}\tilde{y}_{ij}} x_{ik},\quad \mathbf n_{jk} = \mathbf {y}_{i \cdot } -\tilde{\mathbf {y}}_{i \cdot }. \end{aligned}$$

The probability for a molecule to jump is given by the jump coefficient $q_{jk}$. The state change vector has two nonzero components: $n_{jk,j}=-1,\; n_{jk,k}=1$. The diffusion master equation (DME) in a chemical system without reactions is

4 $$\begin{aligned} \partial _tp(\mathbf {y},t)= & {} \displaystyle {\sum _{i=1}^{M}\sum _{j=1}^{N}\sum _{k=1}^{N} q_{jk}(y_{ij}+1)p(\mathbf {y}_{1 \cdot }, \mathbf {y}_{2 \cdot },\ldots , \mathbf {y}_{i \cdot }- \mathbf n_{jk},\ldots , \mathbf {y}_{M \cdot }, t)}\nonumber \\&\displaystyle {-\sum _{i=1}^{M}\sum _{j=1}^{N}\sum _{k=1}^{N} q_{jk}y_{ij}p(\mathbf {y}, t)} \equiv \mathcal {D}p(\mathbf {y}, t), \end{aligned}$$

defining the diffusion operator $\mathcal {D}$. The RDME for the PDF of the state of a system with reactions and diffusion is then

5 $$\begin{aligned} \partial _tp(\mathbf {y},t) = (\mathcal {M}+ \mathcal {D}) p(\mathbf {y}, t). \end{aligned}$$

The jump coefficients $q_{jk}$ are determined by the geometry of $\mathcal {V}$ and the voxels $\mathcal {V}_j$ and the diffusion coefficient $\gamma$. If $\mathcal {V}$ is a rectangle in 2D or a rectangular hexahedron in 3D and the voxels are squares or cubes, then the mesh partitioning $\mathcal {V}$ is Cartesian as in Fig. 1b. When $\mathcal {V}_j$ and $\mathcal {V}_k$ share a boundary and the size of an edge in the mesh is $\Delta x$, we have $q_{jk}=\gamma /\Delta x^2$. If $\mathcal {V}_j\bigcap \mathcal {V}_k=\emptyset$, then $q_{jk}=0$. For a general shape of $\mathcal {V}$, the voxels are defined by an unstructured mesh consisting of triangles (2D) as in Fig. 1a or tetrahedra (3D) in Engblom et al. (2009). If there is an edge $e_{ij}$ between node *j* in $\mathcal {V}_j$ and node *k* in $\mathcal {V}_k$, then $q_{jk}> 0$.

The diffusion equation for $u(\mathbf {x}, t)$ with Neumann boundary conditions in *d* dimensions is

6 $$\begin{aligned} \partial _tu = \gamma \sum _{i=1}^d \frac{\partial ^2u}{\partial x_i^2},\quad \mathbf {x}\in \mathcal {V},\quad \mathbf n\cdot \nabla u=0,\quad \mathbf {x}\in \partial \mathcal {V}, \end{aligned}$$

where $\partial \mathcal {V}$ is the boundary of $\mathcal {V}$ and $\mathbf n$ is the normal of $\partial \mathcal {V}$. The equation is discretized in space by a finite element method in Engblom et al. (2009) to derive $q_{kj}$. Let $u_{ij}$ be the concentration of $y_{ij}$ in $\mathcal {V}_j$ such that $u_{ij}=y_{ij}/V_j$. Then, $\mathbf {u}_{i \cdot }$ for one species *i* satisfies after discretization

7 $$\begin{aligned} \dot{u}_{ij} = \sum _{k\in \mathcal {J}(j)} D_{jk} u_{ik}+D_{jj}u_{ij}, \end{aligned}$$

where $\mathcal {J}(j)$ is the set of nodes connected to node *j* by an edge. It is shown in Engblom et al. (2009) that

8 $$\begin{aligned} D_{jk}=\frac{S_{jk}}{V_j},\quad D_{jj}=-\sum _{k, k\ne j} D_{jk}=\sum _{k\in \mathcal {J}(j)} D_{jk},\quad S_{jk}=S_{kj},\quad S_{jj}=-\sum _{k, k\ne j} S_{jk}.\nonumber \\ \end{aligned}$$

With a mesh of good quality, $S_{jk}\ge 0$ for $j\ne k$, see Meinecke et al. (2016). The relation between the jump coefficients in (3) and the diffusion coefficients in (7) is

9 $$\begin{aligned} q_{kj}=\frac{V_j}{V_k} D_{jk}=\frac{S_{jk}}{V_k}. \end{aligned}$$

To simplify the notation, the assumption here is that the diffusion speed is equal for all species. Otherwise, $q_{kj}, D_{jk},$ and $S_{jk}$ in (9) would be scaled by $\gamma _i/\gamma$ to account for the different diffusion coefficients $\gamma _i$ of the different species *i*.

A numerical solution of (5) is seldom computationally feasible due to the high dimension of $\mathbf {y}\in \mathbb Z^{MN}_+$. Suppose that the lattice for $\mathbf {y}$ has *L* points in each coordinate direction, i.e., $y_{ij}\in [0,1,\ldots ,L]$. Then, the lattice size for $\mathbf {y}$ is $L^{MN}$. A simplification is possible by first splitting the operator $\mathcal {M}+ \mathcal {D}$ into two parts (Hellander et al. 2014; MacNamara and Strang 2016; Strang 1968). Suppose that the solution is known at $t^n$. A timestep $\Delta t$ is chosen, and then, the reaction part (2) is integrated from $t^n$ to $t^{n+1}=t^n+\Delta t$ followed by integration of the diffusion part (4) in

10 $$\begin{aligned} 1.&\partial _tp_1(\mathbf {y},t) = \mathcal {M}p_1(\mathbf {y}, t),\quad t\in [t^n, t^{n+1}],\quad p_1(\mathbf {y}, t^n)=p(\mathbf {y}, t^n),\nonumber \\ 2.&\partial _tp(\mathbf {y},t) = \mathcal {D}p(\mathbf {y}, t),\quad t\in [t^n, t^{n+1}],\quad p(\mathbf {y}, t^n)=p_1(\mathbf {y}, t^{n+1}). \end{aligned}$$

The splitting error in *p* at $t^{n+1}$ is of ${\mathcal O}(\Delta t)$ in (10). Second-order accuracy is obtained by evaluating the reaction equation at half the step at $t^{n+1/2}=t^{n}+0.5\Delta t$, then solving the diffusion Eq. (4) for a full step, and finally taking half a step with (2) (Strang 1968)

11 $$\begin{aligned}&1.~ \partial _tp_1(\mathbf {y},t) = \mathcal {M}p_1(\mathbf {y}, t),\quad t\in [t^n, t^{n+1/2}],\quad p_1(\mathbf {y}, t^n)=p(\mathbf {y}, t^n),\nonumber \\&2.~ \partial _tp_2(\mathbf {y},t) = \mathcal {D}p_2(\mathbf {y}, t),\quad t\in [t^n, t^{n+1}], \quad p_2(\mathbf {y}, t^n)=p_1(\mathbf {y}, t^{n+1/2}),\nonumber \\&3.~ \partial _tp(\mathbf {y},t) = \mathcal {M}p(\mathbf {y}, t),\quad t\in [t^{n+1/2}, t^{n+1}],\quad p(\mathbf {y}, t^{n+1/2})=p_2(\mathbf {y}, t^{n+1}).\qquad \end{aligned}$$

The solution has been advanced from $t^n$ to $t^{n+1}$. The error in $p(\mathbf {y}, t^{n+1})$ is of ${\mathcal O}(\Delta t^2)$.

In (2), the reactions occur independently in every voxel without being influenced by the species in the other voxels. Introduce the ansatz

12 $$\begin{aligned} p(\mathbf {y}_{\cdot 1}, \mathbf {y}_{\cdot 2},\ldots , \mathbf {y}_{\cdot N}, t)=\prod _{j=1}^N p(\mathbf {y}_{\cdot j}, t) \end{aligned}$$

into (2) to arrive at

13 $$\begin{aligned} 0= & {} \partial _tp(\mathbf {y},t) - \left( \sum _{j=1}^N\sum _{r=1}^{R} w_r(\mathbf {y}_{\cdot j}- \mathbf n_r)p(\mathbf {y}_{\cdot 1}, \mathbf {y}_{\cdot 2},\ldots , \mathbf {y}_{\cdot j}- \mathbf n_r, \mathbf {y}_{\cdot N}, t) \right. \nonumber \\&\left. -\sum _{j=1}^N\sum _{r=1}^{R} w_r(\mathbf {y}_{\cdot j})p(\mathbf {y}, t)\right) \nonumber \\= & {} \sum _{j=1}^N \prod _{k=1, k\ne j}^N p(\mathbf {y}_{\cdot k}, t)\left( \partial _tp(\mathbf {y}_{\cdot j}, t)-\left( \sum _{r=1}^{R} w_r(\mathbf {y}_{\cdot j}- \mathbf n_r)p(\mathbf {y}_{\cdot j}- \mathbf n_r, t) \right. \right. \nonumber \\&\left. \left. -\sum _{r=1}^{R} w_r(\mathbf {y}_{\cdot j}, t)p(\mathbf {y}_{\cdot j}, t)\right) \right) . \end{aligned}$$

Hence, for $t\ge t^n$ and $p(\mathbf {y}_{\cdot j}, t^n)$ given, *N* separate solutions of the CME can be computed

14 $$\begin{aligned} \partial _tp(\mathbf {y}_{\cdot j},t)= & {} \sum _{r=1}^{R} w_r(\mathbf {y}_{\cdot j}- \mathbf {m}_r) p(\mathbf {y}_{\cdot j}- \mathbf {m}_r, t)\nonumber \\&- \sum _{r=1}^{R} w_r(\mathbf {y}_{\cdot j})p(\mathbf {y}_{\cdot j}, t),\quad j=1,\ldots ,N, \end{aligned}$$

and then combined in (12) in the first step of (10) (or the first and third steps in (11)). The solution is computed *N* times on a lattice of size $L^M$. This is smaller than the lattice for the full problem but may still be prohibitively large for numerical solution.

In the same manner, the species diffuse independently of each other in the second step in (10) and (11). Insert

15 $$\begin{aligned} p(\mathbf {y}_{1 \cdot }, \mathbf {y}_{2 \cdot },\ldots , \mathbf {y}_{M \cdot }, t)=\prod _{i=1}^M p(\mathbf {y}_{i \cdot }, t) \end{aligned}$$

into (4) and rearrange the terms as in (13) to arrive at *M* separate equations for the diffusion of the species when $t\ge t^n$ and $p(\mathbf {y}_{i\cdot }, t^n)$ is known

16 $$\begin{aligned} \partial _tp(\mathbf {y}_{i \cdot },t)= & {} \sum _{j=1}^{N}\sum _{k=1}^{N}q_{jk}(y_{ij}+1)p(\mathbf {y}_{i \cdot }- \mathbf n_{jk}, t) \nonumber \\&- \sum _{j=1}^{N}\sum _{k=1}^{N} q_{jk}y_{ij}p(\mathbf {y}_{i \cdot }, t), \quad i=1,\ldots ,M. \end{aligned}$$

Since the propensity is linear in $\mathbf {y}$ in (16), there exist analytical solutions to the subproblems with multinomial and Poisson distributions for *p*, see Jahnke and Huisinga (2007), but in practice they are not so useful due to the size $L^N$ of the lattice.

## Linear Noise Approximation

The biochemical systems are assumed to have a scaling with a size parameter $\Omega$ as in van Kampen (2004), Kurtz (1970), Kurtz (1971) where $\Omega \gg 1$. In chemical applications, $\Omega$ can denote a volume or a typical copy number of the species. Then, the copy numbers are rescaled by $\Omega$, $\mathbf {z}=\Omega ^{-1}\mathbf {y}$, and the propensities can be written as

17 $$\begin{aligned} w_{r}(\mathbf {y})= \Omega v_{r}(\Omega ^{-1}\mathbf {y})=\Omega v_{r}(\mathbf {z}),\quad r=1,\ldots ,R. \end{aligned}$$

Equations for approximation of the PDF of a system are derived below. The computational complexity of their solution is polynomial in *M* and *N* instead of exponential as in (5), (14), and (16).

### The Mean Value Equation

Let $m_{ij}$ be the expected value $E[Y_{ij}]$ of the copy number $Y_{ij}$ of species *i* in voxel *j* with a PDF satisfying the master Eq. (2). Multiply (2) by $y_{ij}$ and sum over $\mathbb Z^N_+$. Then, $m_{ij}$ satisfies the equation (Ferm et al. 2008; van Kampen 2004)

18 $$\begin{aligned} \dot{m}_{ij}=\sum _{r=1}^Rn_{ri}E[w_r(\mathbf {Y}_{\cdot j})]. \end{aligned}$$

Suppose that every $w_r$ is linear in $\mathbf {y}$. Then,

$$\begin{aligned} E[w_r(\mathbf {Y}_{\cdot j})]=w_r(E[\mathbf {Y}_{\cdot j}])=w_r(\mathbf {m}_{\cdot j}). \end{aligned}$$

and Eq. (18) are exact for the mean values. If $w_r$ is nonlinear in $\mathbf {y}_{\cdot j}$, then an approximation is

$$\begin{aligned} E[w_r(\mathbf {Y}_{\cdot j})]\approx w_r(\mathbf {m}_{\cdot j}). \end{aligned}$$

With this approximation, we obtain the reaction rate equations

19 $$\begin{aligned} \dot{m}_{ij}=\sum _{r=1}^Rn_{ri}w_r(\mathbf {m}_{\cdot j}). \end{aligned}$$

The mean value equations scaled by the size parameter $\mu _{ij}=m_{ij}/\Omega$ are

20 $$\begin{aligned} \dot{\mu }_{ij}=\sum _{r=1}^Rn_{ri}v_r(\varvec{\mu }_{\cdot j})\equiv \nu _i(\varvec{\mu }_{\cdot j}). \end{aligned}$$

The mean concentration $u_{ij}=\mu _{ij}/V_j$ satisfies

21 $$\begin{aligned} \dot{u}_{ij}=V_j^{-1}\sum _{r=1}^Rn_{ri}v_{r}((\mathbf {u}\mathbf V)_{\cdot j})= V_j^{-1}\nu _i(V_j\mathbf {u}_{\cdot j}). \end{aligned}$$

### The Linear Noise Approximation

The scaled state variable $\mathbf {z}_{\cdot k}$ in voxel *k* is split into a deterministic part $\varvec{\mu }_{\cdot k}$ and a random part $\varvec{\eta }$ by van Kampen in van Kampen (1976, 2004) for the chemical reactions. The random term is assumed to be proportional to $\Omega ^{-1/2}$. The relation between the copy numbers $\mathbf {y}_{\cdot k}$, the scaled copy numbers $\mathbf {z}_{\cdot k}$, the fluctuations $\varvec{\eta }$, and the fluctuations in the concentrations $\varvec{\psi }=V_k^{-1}\varvec{\eta }$ in $\mathcal {V}_k$ is

22 $$\begin{aligned} \mathbf {y}_{\cdot k}=\Omega \mathbf {z}_{\cdot k}=\Omega (\varvec{\mu }_{\cdot k}+\Omega ^{-1/2}\varvec{\eta })=\Omega V_k(\mathbf {u}_{\cdot k}+\Omega ^{-1/2}\varvec{\psi }). \end{aligned}$$

This expansion is inserted into master Eq. (2) with the propensities $v_r$ in (17)

23 $$\begin{aligned} \partial _tp(\Omega \mathbf {z}_{\cdot k}, t)= & {} \sum _r\Omega (v_r(\mathbf {z}_{\cdot k}-\Omega ^{-1}\mathbf n_r) p(\Omega (\mathbf {z}_{\cdot k}-\Omega ^{-1}\mathbf n_r),t)\nonumber \\&- v_r(\mathbf {z}_{\cdot k})p(\Omega \mathbf {z}_{\cdot k}, t)). \end{aligned}$$

Replace *p* in (23) by $\Pi$ in

24 $$\begin{aligned} \Pi (\varvec{\eta }, t)=p(\Omega \varvec{\mu }_{\cdot k}+\Omega ^{1/2}\varvec{\eta }, t)=p(\mathbf {y}_{\cdot k}, t), \end{aligned}$$

and expand the right-hand side of (23) in a Taylor series around $\varvec{\mu }_{\cdot k}$. Terms proportional to $\Omega ^{1/2}$ vanish since $\varvec{\mu }_{\cdot k}$ satisfies (20). If $\Pi$ is the solution to

25 $$\begin{aligned} \Pi _t= & {} \displaystyle {\sum _{r=1}^R\sum _{i=1}^M\left( \Pi v_{r,i} n_{ri} +\sum _{j=1}^M\Pi _{,i} n_{ri} v_{r,j} \eta _j\right) } \displaystyle {+0.5 \sum _{i=1}^M\sum _{j=1}^M W_{ij} \Pi _{,ij},}\nonumber \\ W_{ij}(\varvec{\mu }_{\cdot k})= & {} \displaystyle {\sum _r n_{ri}n_{rj} v_r(\varvec{\mu }_{\cdot k})}, \end{aligned}$$

then terms of ${\mathcal O}(1)$ cancel out. Terms of ${\mathcal O}(\Omega ^{-1/2})$ and smaller are ignored in the expansion. This is the *linear noise approximation* (LNA) for the scaled copy numbers subject to chemical reactions in $\mathcal {V}_k$.

The solution to (25) is the PDF of a normal distribution

26 $$\begin{aligned} \Pi (\varvec{\eta }, t)= & {} \displaystyle {\frac{1}{(2\pi )^{M/2}\sqrt{\det {\varvec{\Sigma }}}} \exp \left( -0.5\sum _{i=1}^M\sum _{j=1}^M\eta _i(\varvec{\Sigma }^{-1})_{ij}\eta _j\right) }, \end{aligned}$$

see Ferm et al. (2008), van Kampen (2004) and (Risken 1996, p. 156). The dimension of $\varvec{\eta }$ is *M* in the CMEs (14) for all *N* voxels. The matrix $\varvec{\Sigma }$ for the covariance between the species *i* and *j* in $\mathcal {V}_k$ is the solution of

27 $$\begin{aligned} \dot{\Sigma }_{ij}=\sum _{l=1}^M\nu _{i,l}\Sigma _{lj}+\sum _{l=1}^M\nu _{j,l}\Sigma _{li}+W_{ij}(\varvec{\mu }_{\cdot k}). \end{aligned}$$

Since $\varvec{\eta }$ is normally distributed with $\mathbf {0}$ mean and covariance $\varvec{\Sigma }$, $\varvec{\eta }\sim \mathcal {N}(\mathbf {0}, \varvec{\Sigma })$, it follows from (22) that $\mathbf {Y}_{\cdot k}, \mathbf {Z}_{\cdot k},$ and the concentration $\mathbf {U}_{\cdot k}=V^{-1}_k\mathbf {Z}_{\cdot k}$ also have normal distributions

28 $$\begin{aligned} \mathbf {Y}_{\cdot k}\sim \mathcal {N}(\Omega \varvec{\mu }_{\cdot k}, \Omega \varvec{\Sigma }),\quad \mathbf {Z}_{\cdot k}\sim \mathcal {N}(\varvec{\mu }_{\cdot k}, \Omega ^{-1}\varvec{\Sigma }), \quad \mathbf {U}_{\cdot k}\sim \mathcal {N}\left( V^{-1}_k\varvec{\mu }_{\cdot k}, \Omega ^{-1}V^{-2}_k\varvec{\Sigma }\right) .\nonumber \\ \end{aligned}$$

The covariance of $\mathbf {U}_{\cdot k}$ in (28) is denoted by $\Omega ^{-1}\varvec{\Xi }=\Omega ^{-1}V^{-2}_k\varvec{\Sigma }$. Then, the differential equation satisfied by $\varvec{\Xi }$ follows from (27)

29 $$\begin{aligned} \dot{\Xi }_{ij}=\sum _{l=1}^M\nu _{i,l}\Xi _{lj}+\sum _{l=1}^M\nu _{j,l}\Xi _{li}+V_k^{-2}W_{ij}(V_k\mathbf {u}_{\cdot k}). \end{aligned}$$

There are *M* nonlinear ODEs to solve in (20) for $\varvec{\mu }_{\cdot k}$ in every voxel $\mathcal {V}_k$. The covariance matrix $\varvec{\Sigma }$ is symmetric, and we have to solve $(M+1)M/2$ linear ODEs in (27) and (29). The structure of this equation is the same also for other approximations of the CME, e.g., EMRE in Smith et al. (2017). The accuracy of mean value Eq. (20) is improved in Ferm et al. (2008) by adding a term which is linear in the covariance.

### The Diffusion Equation

The notation is simplified if we assume here that there is only one species, $M=1$, but many voxels, $N>1$. If $M>1$, then the diffusion of the other species is treated separately in the same manner, see (16). In diffusion master Eq. (16), the propensity to jump from voxel *k* to *j* is linear in $\mathbf {y}\in \mathbb Z_+^N$ with $w_r(\mathbf {y})=q_{kj}y_{k}$ and $v_r(\mathbf {z})=q_{kj}z_{k}$. The linearity implies that there are explicit expressions for the mean value equations, $\nu _{i,k}$ in (27), and $W_{ij}$ in (25).

The equations for the scaled mean values are obtained from (8), (9), and (20)

30 $$\begin{aligned} \dot{\mu }_{j}=\sum _{k=1}^N S_{jk}\mu _k/V_k, \quad j=1,\ldots ,N. \end{aligned}$$

The diffusion equation for the mean concentration is derived from (21)

31 $$\begin{aligned} \dot{u}_{j}=V_j^{-1}\sum _{l=1}^Nq_{lj}V_l u_l= \sum _{l=1}^NS_{jl}u_l/V_j=\sum _{l=1}^ND_{jl}u_l, \quad j=1,\ldots ,N, \end{aligned}$$

cf. (7).

The equation for the covariance $\varvec{\Sigma }$ (27) between voxels *i* and *j* depends on $\nu _{j,k}$ and $W_{ij}$ in (25). The derivative $\nu _{i,k}$ in (27) and (29) is by (8) and (9)

32 $$\begin{aligned} \nu _{i,k}= & {} \displaystyle {\sum _{r=1}^Rn_{ri}w_{r,k}(\mathbf {y})=q_{ki}=S_{ik}/V_k,\; k\ne i,}\nonumber \\ \nu _{i,i}= & {} \displaystyle {\sum _{r=1}^Rn_{ri}w_{r,i}(\mathbf {y})=\sum _{j\in \mathcal {J}(i)}-q_{ij}=-\sum _{j\in \mathcal {J}(i)}S_{ji}/V_i=S_{ii}/V_i,} \end{aligned}$$

since $n_{ri}=1$ for the jump from *k* to *i* and $n_{ri}=-1$ for all jumps from *i* to every *j* connected to node *i* by an edge $e_{ij}$. Let $\mathcal {E}$ be the set of all edges in the mesh. The state change vector on edge $e_{ij}$ for a jump from *i* to *j* is $\mathbf n_{ij}$ with the nonzero components $n_{ij;i}=-1$ and $n_{ij;j}=1$. The contribution to $\mathbf W$ in (27) from $e_{ij}$ jumps in two directions: $i\rightarrow j$ and $j\rightarrow i$. Hence, for all edges

33 $$\begin{aligned} \mathbf W=\displaystyle {\sum _{e_{ij}\in \mathcal {E}} \frac{S_{ij}}{V_j}\mu _j \mathbf n_{ji}\mathbf n_{ji}^T + \frac{S_{ji}}{V_i}\mu _i \mathbf n_{ij}\mathbf n_{ij}^T } =\displaystyle {\sum _{e_{ij}\in \mathcal {E}} S_{ij}\left( \frac{\mu _j}{V_j}+\frac{\mu _i}{V_i}\right) \mathbf n_{ij}\mathbf n_{ij}^T}. \end{aligned}$$

The nonzero elements of $\mathbf {N}_{ij}=\mathbf n_{ij}\mathbf n_{ij}^T$ are $N_{ij;ii}=N_{ij;jj}=1$ and $N_{ij;ij}=N_{ij;ji}=-1$. Therefore, the elements of the symmetric $\mathbf W$ are

34 $$\begin{aligned} W_{ij}=-S_{ij}\left( \frac{\mu _j}{V_j}+\frac{\mu _i}{V_i}\right) ,\quad j\ne i,\quad W_{ii}=\sum _{j\in \mathcal {J}(i)} S_{ij}\left( \frac{\mu _j}{V_j}+\frac{\mu _i}{V_i}\right) . \end{aligned}$$

The random component of the concentrations $\varvec{\psi }=\mathbf V^{-1}\varvec{\eta }$ is normally distributed $\mathcal {N}(\mathbf {0}, \mathbf V^{-1}\varvec{\Sigma }\mathbf V^{-1})$. The equation for $\varvec{\Xi }=\mathbf V^{-1}\varvec{\Sigma }\mathbf V^{-1}$ is derived from (27)

35 $$\begin{aligned} \dot{\Xi }_{ij}=\sum _{l=1}^MV_i^{-1}\nu _{i,l}V_l \Xi _{lj}+\sum _{l=1}^MV_j^{-1}\nu _{j,l}V_l\Xi _{li}+V_i^{-1}W_{ij}(\mathbf V\mathbf {u})V_j^{-1}. \end{aligned}$$

The coefficients in (35) multiplying $\varvec{\Xi }$ are

36 $$\begin{aligned} V_i^{-1}\nu _{i,l}V_l=S_{il}/V_i=D_{il},\; l\ne i,\quad V_i^{-1}\nu _{i,i}V_i=S_{ii}/V_i=D_{ii}. \end{aligned}$$

In (35), $\mathbf W$ is scaled by $\mathbf V$

37 $$\begin{aligned} V_i^{-1}W_{ij}V_j^{-1}= & {} \displaystyle {-V_i^{-1}S_{ij}V_j^{-1}(u_i+u_j)=-D_{ij}V_j^{-1}(u_i+u_j),\; j\ne i,}\nonumber \\ V_i^{-1}W_{ii}V_i^{-1}= & {} \displaystyle {2\sum _{j\in \mathcal {J}(i)} V_i^{-1}S_{ij}V_i^{-1}u_i=-2V_i^{-1}S_{ii}V_i^{-1}u_i}\nonumber \\= & {} \displaystyle {-2D_{ii}V_i^{-1}u_i.} \end{aligned}$$

Then, the scaled $\mathbf W$-term in (37) can be written in a symmetric form

38 $$\begin{aligned} V_i^{-1}W_{ij}V_j^{-1}= & {} -D_{ij}V_j^{-1}(u_i+u_j)=-S_{ij}V_i^{-1}V_j^{-1}(u_i+u_j)\nonumber \\= & {} -\left( u_iD_{ij}V_j^{-1}+u_jD_{ji}V_i^{-1}\right) . \end{aligned}$$

The covariance equation corresponding to (29) for diffusion is by (35), (36), and (38)

39 $$\begin{aligned} \dot{\Xi }_{ij}= & {} \displaystyle {\sum _{l=1}^N D_{il}\Xi _{lj} +\sum _{l=1}^N D_{jl}\Xi _{li}-f_{ij},}\nonumber \\ f_{ij}= & {} \displaystyle {u_iD_{ij}V_j^{-1}+u_jD_{ji}V_i^{-1},} \quad i,j=1,\ldots ,N. \end{aligned}$$

The copy numbers $\mathbf {Y}_{k \cdot }$ and $\mathbf {Z}_{k \cdot }$ are normally distributed as in (28). The covariance of the concentrations in space of a species *k*, $\mathbf {U}_{k \cdot }$, is $\Omega ^{-1}\varvec{\Xi }$ and $\mathbf {U}_{k \cdot }\sim \mathcal {N}(\mathbf {u}_{k \cdot }, \Omega ^{-1}\varvec{\Xi })$.

In the stationary equation, $\dot{\Xi }_{ij}=0$ in (39) and it is a Lyapunov equation. A stationary solution of (31) is $u_i=\mathrm{const.}$ and of (39) is

40 $$\begin{aligned} \Xi _{ij}=u_iV_i^{-1}\delta _{ij}, \end{aligned}$$

where $\delta _{ij}$ is the Kronecker delta. If the initial data $\Xi _{ij}(0)$ are symmetric, then the solution to (39) is symmetric for all $t>0$. At the stationary solution of (40)

$$\begin{aligned} \varvec{\Sigma }=\mathbf V\varvec{\Xi }\mathbf V=\mathrm{diag}(\mathbf V\mathbf {u}),\quad \varvec{\mu }=\mathbf V\mathbf {u}, \end{aligned}$$

where $\mathrm{diag}(\mathbf {x})$ is a diagonal matrix with $x_i$ in the diagonal. Thus, the stationary distribution of the copy numbers in the voxels $\mathbf {Y}_{k \cdot }$ for species *k* follows from (28)

41 $$\begin{aligned} \mathbf {Y}_{k \cdot }\sim \mathcal {N}(\Omega \mathbf V\mathbf {u}, \Omega \,\mathrm{diag}(\mathbf V\mathbf {u})). \end{aligned}$$

The stationary copy numbers in different voxels are uncorrelated, have a multivariate normal distribution and are therefore independent, and are approximately Poisson distributed since $Y_{ki}\sim \mathcal {N}(\Omega V_iu_i, \Omega V_iu_i)$ with equal mean and variance. If $\Xi _{ij}(0)=0$, then the time-dependent solutions to (39) will be proportional to $u_i/V_i$ and the mean and the covariance of $Y_{ij}$ are both proportional to $\Omega V_i u_i$. The distributions of the solution to the DME in (4) are discussed in Engblom et al. (2017) based on the theory for linear propensities in Jahnke and Huisinga (2007). Their distributions are either multinomial, Poisson, or a combination. The stationary distribution is multinomial according to Anderson et al. (2010) and approximately Poissonian (Engblom et al. 2017).

The components of the solution $\mathbf {u}$ of (31) are the node values of the finite element approximation of $u(\mathbf {x}, t)$ solving diffusion Eq. (6) for one species. Let $\Xi (\mathbf {x}_1, \mathbf {x}_2, t)$ be the covariance between the solutions at the coordinates $\mathbf {x}_1, \mathbf {x}_2\in \mathbb R^d$. Then, $\Xi _{ij}$ in (39) can be interpreted as the value of $\Xi (\mathbf {x}_1, \mathbf {x}_2, t)$ at the nodes at $\mathbf {x}_{1i}$ and $\mathbf {x}_{2j}$. The coefficient $D_{ij}$ in $f_{ij}$ in (39) is negative when $\mathbf {x}_{1i}=\mathbf {x}_{2j}$, and positive when $\mathbf {x}_{1i}\ne \mathbf {x}_{2j}$. When $D_{ij}$ is nonzero, the difference $\varvec{\xi }_{ij}=\frac{1}{\sqrt{2}}(\mathbf {x}_{1i}-\mathbf {x}_{2j})$ is small. On a regular mesh with a typical length of an edge equal to $\Delta x$, the positive weight $D_{ij}$ depends approximately only on $r_{ij}=\Vert \varvec{\xi }_{ij}\Vert$ and $V_i$ varies smoothly with a typical size *V*. On such a mesh, $D_{ij}\propto \Delta x^{-2}$ and $u_iD_{ij}V_j^{-1}$ is approximated here by a continuous function $u(\mathbf {x}_1, t)\varphi (r_{ij})$ with

42 $$\begin{aligned} \varphi (r)=-\frac{\gamma }{\Delta x^2V}\exp \left( \frac{-r^2}{\sigma ^2}\right) \cos (\pi \omega r), \end{aligned}$$

and the solution $u(\mathbf {x}, t)$ to (6). The scalings $\sigma$ and $\omega$ are chosen such that $\sigma \propto \Delta x$ and $\omega =\Delta x^{-1}$. When $r=0$ then $D_{ii}V_i\approx \varphi (0)=-\gamma /\Delta x^2V$ and when $r=\Delta x$ we have

$$\begin{aligned} D_{ij}V_j^{-1}\approx \varphi (\Delta x)=\frac{\gamma }{\Delta x^2V} \exp \left( -\left( \frac{\Delta x}{\sigma }\right) ^2\right) <|\varphi (0)|. \end{aligned}$$

Then, the continuous equation corresponding to discrete Eq. (39) is

43 $$\begin{aligned} \partial _t\Xi =\gamma \sum _{i=1}^d \frac{\partial ^2 \Xi }{\partial x_{1i}^2}+\frac{\partial ^2 \Xi }{\partial x_{2i}^2}-(u(\mathbf {x}_1, t)+u(\mathbf {x}_2, t))\varphi (r). \end{aligned}$$

If exact initial conditions of the distribution of molecules are known, then $\Xi (\mathbf {x}_1, \mathbf {x}_2, 0)=0$.

Let $u_\infty (\mathbf {x})$ be the stationary solution to the diffusion equation. Then, one can show that an approximate stationary solution to (43) is

44 $$\begin{aligned} \Xi _{\infty }(\mathbf {x}_1, \mathbf {x}_2)=\frac{u_{\infty }(\mathbf {x}_1)}{V}\exp \left( -\frac{\Vert \mathbf {x}_1-\mathbf {x}_2\Vert ^2}{2\sigma ^2}\right) , \end{aligned}$$

As $\Delta x\rightarrow 0$, this solution approaches $u_\infty (\mathbf {x}_1)\delta (\Vert \mathbf {x}_1-\mathbf {x}_2\Vert )$ where $\delta$ is the Dirac measure. The solution in (40) to discrete Eq. (39) is similar to (44).

## Analysis of the Covariance Equation

A property of the continuous approximation $\Xi (\mathbf {x}_1, \mathbf {x}_2, t)$ of the covariance in (43) is derived in this section. We show that $\Xi$ decays exponentially when $\Vert \mathbf {x}_1-\mathbf {x}_2\Vert$ grows, indicating that the discrete variance $\Xi _{ij}$ in (39) is small when $\Vert \mathbf {x}_{1i}-\mathbf {x}_{2j}\Vert > \Delta x$.

Consider (43) in free space $\mathbf {x}_1, \mathbf {x}_2\in \mathbb R^{d}$ and for $t\ge 0$ with initial data $\Xi (\mathbf {x}_1, \mathbf {x}_2, 0)$. The concentration $u(\mathbf {x}_1, t)$ is nonnegative and is assumed to be bounded by $C_u$ for all $\mathbf {x}_1$ and $t\ge 0$. Introduce a change of variables

45 $$\begin{aligned} \left( \begin{array}{c} \xi _{1j}\\ \xi _{2j} \end{array}\right) =\frac{1}{\sqrt{2}}\left( \begin{array}{cc} 1&{}-1\\ 1&{}1\end{array}\right) \left( \begin{array}{c} x_{1j}\\ x_{2j}\end{array}\right) ,\quad j=1,\ldots ,d. \end{aligned}$$

The diffusion equation in (43) is in the new variables

46 $$\begin{aligned} \partial _t\Xi =\gamma \sum _{j=1}^d \frac{\partial ^2 \Xi }{\partial \xi _{1j}^2}+\frac{\partial ^2 \Xi }{\partial \xi _{2j}^2}-f(\varvec{\xi }_1, \varvec{\xi }_2, t)\varphi (\Vert \varvec{\xi }_1\Vert ). \end{aligned}$$

Here $f(\varvec{\xi }_1, \varvec{\xi }_2, t)=u(\mathbf {x}_1, t)+u(\mathbf {x}_2, t)$ is nonnegative and bounded by $2C_u$. The factor $\varphi$ in the source term vanishes quickly when $\Vert \varvec{\xi }_1\Vert$ increases.

With the fundamental solution of the diffusion equation in 2*d* dimensions (Evans 2010; Stakgold 2000), the solution of (46) can be written as a sum of two integrals depending on the initial data and the source term

47 $$\begin{aligned} \Xi (\varvec{\xi }_1, \varvec{\xi }_2, t)= I_\mathrm{ini}+I_\mathrm{src}, \end{aligned}$$

where

48 $$\begin{aligned} I_\mathrm{ini}= & {} \int _{\mathbb R^{d}}\int _{\mathbb R^{d}}\frac{1}{(4\pi \gamma t)^{d}} \exp \left( -\left( \Vert \varvec{\xi }_1-\varvec{\zeta }_1\Vert ^2+\Vert \varvec{\xi }_2-\varvec{\zeta }_2\Vert ^2\right) /4\gamma (t)\right) \nonumber \\&\Xi (\varvec{\zeta }_1, \varvec{\zeta }_2, 0)\, \mathrm{d}\varvec{\zeta }_1\, \mathrm{d}\varvec{\zeta }_2, \end{aligned}$$

and

49 $$\begin{aligned} I_\mathrm{src}= & {} \displaystyle {-\int _0^t\int _{\mathbb R^{d}}\int _{\mathbb R^{d}}\frac{\gamma }{(4\pi \gamma (t-s))^{d}} \exp \left( -\left( \Vert \varvec{\xi }_1-\varvec{\zeta }_1\Vert ^2+\Vert \varvec{\xi }_2-\varvec{\zeta }_2\Vert ^2\right) /(4\gamma (t-s))\right) }\nonumber \\&\displaystyle {\cdot \frac{f(\varvec{\zeta }_1, \varvec{\zeta }_2, t)}{\Delta x^2V}\cos (\omega \Vert \varvec{\zeta }_{1}\Vert )\prod _{j=1}^d \exp (-\zeta _{1j}^2/\sigma ^2)\, \mathrm{d}\varvec{\zeta }_1\,\mathrm{d}\varvec{\zeta }_2\,\mathrm{d}s.} \end{aligned}$$

The integral with the source term is bounded by

50 $$\begin{aligned} |I_\mathrm{src}|\le & {} \displaystyle {\frac{2C_u\gamma }{\Delta x^2V}\int _0^t\frac{1}{(4\pi \gamma (t-s))^{d}}\int _{\mathbb R^{d}} \exp \left( -\Vert \varvec{\xi }_2-\varvec{\zeta }_2\Vert ^2/(4\gamma (t-s))\right) \, \mathrm{d}\varvec{\zeta }_2}\nonumber \\&\displaystyle \cdot \int _{\mathbb R^{d}}|\cos (\omega \Vert \varvec{\zeta }_{1}\Vert )|\prod _{j=1}^{d}\exp \left( -(\xi _{1j}- \zeta _{1j})^2/(4\gamma (t-s))\right) \nonumber \\&\times \exp \left( -\zeta _{1j}^2/\sigma ^2\right) \, \mathrm{d}\varvec{\zeta }_{1}\,\mathrm{d}s. \end{aligned}$$

The spatial integral $I_{d2}$ over $\varvec{\zeta }_2\in \mathbb R^d$ in (50) is

51 $$\begin{aligned} I_{d2}(t-s)= & {} \prod _{j=1}^{d} \int _\mathbb R\exp (-(\xi _{2j}-\zeta )^2/(4\gamma (t-s)))\, \mathrm{d}\zeta =\prod _{j=1}^{d} 2\sqrt{\pi \gamma (t-s)}\nonumber \\= & {} (4\pi \gamma (t-s))^{d/2}. \end{aligned}$$

The integral $I_{d1}$ of the product over $\varvec{\zeta }_1\in \mathbb R^d$ in (50) is

52 $$\begin{aligned}&I_{d1}(t-s,\sigma )\nonumber \\&\quad =\displaystyle {\int _{\mathbb R^{d}} \prod _{j=1}^{d}\exp \left( -(\xi _{1j}-\zeta _{1j})^2/ (4\gamma (t-s))\right) \exp \left( -\zeta _{1j}^2/\sigma ^2\right) \, \mathrm{d}\varvec{\zeta }_{1}}\nonumber \\&\quad =\displaystyle {\prod _{j=1}^{d} \int _\mathbb R\exp \left( -(\xi _j-\zeta )^2/(4\gamma (t-s))\right) \exp \left( -\zeta ^2/\sigma ^2\right) \, \mathrm{d}\zeta =\prod _{j=1}^{d} I_{1j}(\sigma )}.\nonumber \\ \end{aligned}$$

With $\tau =4\gamma (t-s)$ and $\alpha =\tau ^{-1}+\sigma ^{-2}$, we have

53 $$\begin{aligned} I_{1j}(\sigma )= & {} \displaystyle {\int _\mathbb R\exp \left( -(\xi _{1j}-\zeta )^2/\tau -\zeta ^2/\sigma ^2\right) \, \mathrm{d}\zeta }\nonumber \\= & {} \displaystyle {\exp \left( -\xi _{1j}^2/\tau +\xi _{1j}^2/\alpha \tau ^2\right) \int _\mathbb R\exp \left( -\alpha (\zeta -\xi _{1j}/\alpha \tau )^2\right) \, \mathrm{d}\zeta }\nonumber \\= & {} \displaystyle {\frac{\exp \left( -\xi _{1j}^2/(\tau +\sigma ^2)\right) }{\sqrt{\alpha }}\int _\mathbb R\exp (-z^2)\, \mathrm{d}z}\nonumber \\= & {} \displaystyle {\frac{\sigma \sqrt{\pi \tau }}{\sqrt{\tau +\sigma ^2}} \exp \left( -\xi _{1j}^2/(\tau +\sigma ^2)\right) }. \end{aligned}$$

Using (53), (52), and (51), a bound on $I_\mathrm{src}$ in (50) is

54 $$\begin{aligned} |I_\mathrm{src}| \displaystyle\le & {} \frac{2C_u}{\Delta x^2V}\int _0^t \frac{\gamma }{(4\pi \gamma (t-s))^{d}}I_{d1}(t-s, \sigma )I_{d2}(t-s) \,\mathrm{d}s\nonumber \\ \displaystyle= & {} \frac{2C_u}{\Delta x^2V}\int _0^{4\gamma t} \frac{(\pi \tau )^{d/2}}{4(\pi \tau )^{d}} \prod _{j=1}^dI_{1j}(\sigma ) \,\mathrm{d}\tau \nonumber \\ \displaystyle= & {} \frac{C_u\sigma ^d}{2\Delta x^2V}\int _0^{4\gamma t}\frac{\exp (-\Vert \varvec{\xi }_1\Vert ^2/(\tau +\sigma ^2))}{(\tau +\sigma ^2)^{d/2}}\, \mathrm{d}\tau \nonumber \\ \displaystyle\le & {} \frac{C_u\sigma ^d}{2\Delta x^2V}\exp \left( -\Vert \varvec{\xi }_1\Vert ^2/(4\gamma t+\sigma ^2)\right) \int _0^{4\gamma t}\frac{\mathrm{d}\tau }{(\tau +\sigma ^2)^{d/2}}\nonumber \\= & {} \left\{ \begin{array}{ll} \displaystyle {\frac{C_u\sigma ^2}{2\Delta x^2V}\exp (-\Vert \varvec{\xi }_1\Vert ^2/(4\gamma t+\sigma ^2))\log {\left( 1+\frac{4\gamma t}{\sigma ^2}\right) }},&{} d=2,\\ \displaystyle {\frac{C_u\sigma ^2}{\Delta x^2V(d-2)}\exp (-\Vert \varvec{\xi }_1\Vert ^2/(4\gamma t+\sigma ^2))\left( 1-1/\left( 1+\frac{4\gamma t}{\sigma ^2}\right) ^{d/2-1}\right) },&{} \begin{array}{c} d=1\\ d\ge 3.\end{array} \end{array}\right. \nonumber \\ \end{aligned}$$

Assume that the initial data are localized close to $\varvec{\xi }_1=0$ such that $|\Xi (\mathbf {x}_1, \mathbf {x}_2, 0)|\le \Xi _0\exp (-\Vert \varvec{\xi }_1\Vert ^2/\chi ^2)$ for some $\chi >0$. A bound on the integral in (47) due to the initial data is then

55 $$\begin{aligned} |I_\mathrm{ini}|\le & {} \displaystyle {\frac{\Xi _0}{(4\pi \gamma t)^{d}}\int _{\mathbb R^d}\exp \left( -\left( \Vert \varvec{\xi }_1-\varvec{\zeta }_1\Vert ^2/4\gamma t+\Vert \varvec{\zeta }_1\Vert ^2/\chi ^2\right) \right) \, \mathrm{d}\varvec{\zeta }_1}\nonumber \\&\displaystyle {\cdot \int _{\mathbb R^d}\exp \left( -\Vert \varvec{\xi }_2-\varvec{\zeta }_2\Vert ^2/4\gamma t\right) \, \mathrm{d}\varvec{\zeta }_2 =\frac{\Xi _0}{(4\pi \gamma t)^{d}}I_{d1}(t,\chi )I_{d2}(t)}\nonumber \\= & {} \displaystyle {\frac{\Xi _0}{(4\pi \gamma t)^{d}}\prod _{j=1}^d I_{1j}(\chi ) \cdot (4\pi \gamma t)^{d/2}}\nonumber \\ \displaystyle= & {} \Xi _0\exp \left( -\Vert \varvec{\xi }_1\Vert ^2/(4\gamma t+\chi ^2)\right) /\left( 1+\frac{4\gamma t}{\chi ^2}\right) ^{d/2}. \end{aligned}$$

Hence, a bound on the covariance solution in (47) is obtained by (54) and (55).

The assumptions and conclusions are summarized in a theorem:

### Theorem 1

Assume that $|u(\mathbf {x}, t)|\le C_u$ and that the initial data satisfy

$$\begin{aligned} |\Xi (\mathbf {x}_1, \mathbf {x}_2, 0)|\le \Xi _0\exp (-\Vert \varvec{\xi }_1\Vert ^2/\chi ^2). \end{aligned}$$

The relations between the $\mathbf {x}$ and $\varvec{\xi }$ coordinates are $\varvec{\xi }_1=\frac{1}{\sqrt{2}}(\mathbf {x}_1-\mathbf {x}_2),\; \varvec{\xi }_2=\frac{1}{\sqrt{2}}(\mathbf {x}_1+\mathbf {x}_2)$. Then, the solution of (46) with $\varphi$ defined by (42) for $t>0$ is bounded by

56 $$\begin{aligned} |\Xi (\mathbf {x}_1, \mathbf {x}_2, t)|\le & {} \displaystyle {\frac{C_u\sigma ^2}{\Delta x^2V}f_d\left( 1+\frac{4\gamma t}{\sigma ^2}\right) \exp \left( -(\Vert \varvec{\xi }_1\Vert /\sigma )^2/(1+4\gamma t/\sigma ^2)\right) }\nonumber \\&+\displaystyle {\frac{\Xi _0}{(1+4\gamma t/\chi ^2)^{d/2}}\exp \left( -(\Vert \varvec{\xi }_1\Vert /\chi )^2/(1+4\gamma t/\chi ^2)\right) }, \end{aligned}$$

where

57 $$\begin{aligned} f_d(\zeta )=\frac{1}{2}\log (\zeta ),\quad d=2,\quad f_d(\zeta )=\frac{1}{d-2}\left( 1-\frac{1}{\zeta ^{d/2-1}}\right) ,\quad d=1,\quad d\ge 3.\nonumber \\ \end{aligned}$$

$$\square $$

The function $f_d$ depends on the dimension *d* and is 0 at $t=0$. The first term in (56) is proportional to $C_u/V=\sup u/V$ since $\sigma \propto \Delta x$. The solution in (56) decays exponentially in $\Vert \varvec{\xi }_1\Vert =\frac{1}{\sqrt{2}}\Vert \mathbf {x}_1-\mathbf {x}_2\Vert$ for a fixed *t* in all dimensions and is small when $\Vert \varvec{\xi }_1\Vert >\sigma \propto \Delta x$. For a given $\varvec{\xi }_1$, the first term in (56) increases slowly with *t* in 1 and 2 dimensions and is bounded by $C_u\sigma ^2/\Delta x^2V(d-2)$ when $d\ge 3$. The second term in (56) decreases when $t\ge 0.5\Vert \varvec{\xi }_1\Vert ^2-0.25\chi ^2$ for $d=1$ and for all $t\ge 0$ when $d\ge 2$.

Our bounded domain $\mathcal {V}$ for $\mathbf {x}_1$ and $\mathbf {x}_2$ has a boundary that is not taken into account in (56). The bound on $\Xi$ is a good estimate when the main part of the solution is away from the boundary, e.g., when *t* is not too large and $u(\mathbf {x}, t)$ is nonzero only in the middle of $\mathcal {V}$.

Since (43) is a continuous approximation of (39) we expect the discrete variance $\Xi _{ij}$ to behave in a similar way and be negligible when the nodes *i* and *j* are not neighbors and not directly connected by an edge in the mesh. This property will be exploited in the algorithm in the next section.

## Algorithm

The algorithm to compute the solution to the LNA for both reactions and diffusion is based on the operator splitting in Sect. 2, the derivations in Sects. 3.2 and 3.3, and Theorem 1 in Sect. 4.

The mean value equation in (21) is added to the diffusion equation in (31) to obtain the reaction–diffusion equation for the concentration $u_{ik}$ of species *i* in voxel *k* with *MN* components

58 $$\begin{aligned} \dot{u}_{ik}=V_k^{-1}\nu _i(V_k\mathbf {u}_{\cdot k})+\sum _{\beta =1}^ND_{k\beta }u_{i\beta },\quad i=1,\ldots ,M,\quad j=1,\ldots ,N. \end{aligned}$$

The covariance between the concentrations of the species *i* and *j* in voxels *k* and *l* is written as ${\Xi }_{ij;kl}$ and has $M^2N^2$ components. The equation satisfied by the covariance is obtained from (29) and (39)

59 $$\begin{aligned} \dot{\Xi }_{ij;kl}= & {} \displaystyle {\sum _{\alpha =1}^M\nu _{i,\alpha }\Xi _{\alpha j;kl}+\sum _{\alpha =1}^M\nu _{j,\alpha }\Xi _{\alpha i;kl}+\delta _{kl}V_k^{-2}W_{ij}(V_k\mathbf {u}_{\cdot k})}\nonumber \\&\displaystyle {+\sum _{\beta =1}^N D_{k \beta }\Xi _{ij;\beta l} +\sum _{\beta =1}^N D_{l \beta }\Xi _{ij; \beta k}-\delta _{ij}\left( u_{ik}D_{kl}V^{-1}_l+u_{jl}D_{lk}V^{-1}_k\right) ,}\nonumber \\&i,j=1,\ldots ,M,\quad k,l=1,\ldots ,N. \end{aligned}$$

The reaction source term vanishes if the concentrations are from different voxels, since there is no reaction between molecules in separate voxels. The diffusion source term is zero if the species are different since a diffusion event occurs when the same species changes location between adjacent voxels by a jump.

The equations for the mean and the covariance (58) and (59) are solved by splitting the operator on the right-hand side and advancing the solution one timestep from $t^n$ to $t^{n+1}=t^n+\Delta t$ as in (10) with $u_{ik}(t^n)$ and $\Xi _{ij;kl}(t^n)$ as initial data, cf. (10). The algorithm is

### Algorithm 1

1. 60 $$\begin{aligned} \dot{\tilde{u}}_{ik}=V_k^{-1}\nu _i(V_k\tilde{\mathbf {u}}_{\cdot k}),\quad t\in [t^n, t^{n+1}], \quad \tilde{u}_{ik}(t^n)=u_{ik}(t^n) \end{aligned}$$
2. 61 $$\begin{aligned} \dot{\tilde{\Xi }}_{ij;kk}= & {} \sum _{\alpha =1}^M\nu _{i,\alpha }\tilde{\Xi }_{\alpha j;kk}+\sum _{\alpha =1}^M\nu _{j,\alpha }\tilde{\Xi }_{\alpha i;kk}+V_k^{-2}W_{ij}(V_k\tilde{\mathbf {u}}_{\cdot k}),\nonumber \\&t\in [t^n, t^{n+1}],\quad \tilde{\Xi }_{ij;kk}(t^n)=\Xi _{ij;kk}(t^{n}) \end{aligned}$$
3. 62 $$\begin{aligned} \dot{\tilde{\Xi }}_{ij;kl}= & {} \sum _{\alpha =1}^M\nu _{i,\alpha }\tilde{\Xi }_{\alpha j;kl}+\sum _{\alpha =1}^M\nu _{j,\alpha }\tilde{\Xi }_{\alpha i;kl},\quad k\ne l,\nonumber \\&t\in [t^n, t^{n+1}],\quad \tilde{\Xi }_{ij;kl}(t^n)={\Xi }_{ij;kl}(t^{n}) \end{aligned}$$
4. 63 $$\begin{aligned} \dot{u}_{ik}=\sum _{\beta =1}^ND_{k\beta }u_{i\beta },\quad t\in [t^n, t^{n+1}], \quad u_{ik}(t^n)=\tilde{u}_{ik}(t^{n+1}) \end{aligned}$$
5. 64 $$\begin{aligned} \dot{\Xi }_{ii;kl}= & {} \sum _{\beta =1}^N D_{k \beta }\Xi _{ii;\beta l} +\sum _{\beta =1}^N D_{l \beta }\Xi _{ii; \beta k}-\left( {u}_{ik}D_{kl}V^{-1}_l+{u}_{il}D_{lk}V^{-1}_k\right) ,\nonumber \\&t\in [t^n, t^{n+1}],\quad \Xi _{ii;kl}(t^n)=\tilde{\Xi }_{ii;kl}(t^{n+1}) \end{aligned}$$
6. 65 $$\begin{aligned} \dot{\Xi }_{ij;kl}= & {} \sum _{\beta =1}^N D_{k \beta }\Xi _{ij;\beta l} +\sum _{\beta =1}^N D_{l \beta }\Xi _{ij; \beta k},\quad i\ne j,\nonumber \\&t\in [t^n, t^{n+1}],\quad \Xi _{ij;kl}(t^n)=\tilde{\Xi }_{ij;kl}(t^{n+1}) \end{aligned}$$

The discretization error in $u_{ik}(t^{n+1})$ and $\Xi _{ij;kl}(t^{n+1})$ will be of ${\mathcal O}(\Delta t)$. The ODEs in steps 1, 2 and 3 update *u* and $\Xi$ in a voxel (steps 1, 2) and $\Xi$ between two adjacent voxels (step 3) due to the reactions as in step 1 of (10) and (14). In the ODEs in steps 4, 5 and 6, *u* and $\Xi$ change due to diffusion between voxels without any influence of the other species as in step 2 of (10) and (16). A more accurate splitting than in Algorithm 1 with an error of ${\mathcal O}(\Delta t^2)$ is possible in the same manner as in (11).

It follows from Theorem 1 that if $\Xi _{ij;kl}(t^{n})$ decays rapidly when the nodes $\mathbf {x}_k$ and $\mathbf {x}_l$ are separated then this property is preserved in $\tilde{\Xi }_{ij;kl}(t^{n+1})$ where $C_u> 0$ in (56) in step 2 and $C_u=0$ without the source term in step 3. Using the same arguments in steps 5 and 6, we find that if $\Xi _{ij;kl}(t^{n})$ decays rapidly when $\Vert \mathbf {x}_k-\mathbf {x}_l\Vert$ increases, then after one timestep $\Xi _{ij;kl}(t^{n+1})$ also decays rapidly in $\Vert \mathbf {x}_k-\mathbf {x}_l\Vert$.

Supported by the analysis in Sect. 4, we assume that $\Xi _{ij;kl}$ is negligible when node *l* and node *k* are not neighbors, $l \notin \mathcal {J}(k)$, and we let $\hat{\Xi }_{ij;kl}=0$ in a sparse approximation of ${\Xi }_{ij;kl}$. Then, only elements of $\Xi _{ij;kl}$ when $k=l$ and $l\in \mathcal {J}(k)$ need to be stored and updated in $\hat{\Xi }_{ij;kl}$ by Algorithm 1. The sparsity (or nonzero) pattern of $\hat{\Xi }_{ij;kl}$ for each pair *i*, *j* is the same as that of $\mathbf {S}$ and $\mathbf {D}$ in (8) since $S_{kl}$ and $D_{kl}$ are nonzero only on the diagonal and if nodes the *k* and *l* are neighbors connected by an edge in the mesh and $l\in \mathcal {J}(k)$. Moreover, $\Xi _{ij;kl}$ is symmetric in both *i* and *j* and *k* and *l*. With *M* different species and *N* voxels, $\Xi _{ij;kl}$ in general has $\frac{1}{2}MN(MN+1)$ different elements but $\hat{\Xi }_{ij;kl}$ has only $C_{d}M^2N$ nonzero elements that are necessary to store taking the symmetry into account. The coefficient $C_{d}$ depends on the dimension and the structure of the mesh. In a Cartesian mesh, $C_{d}=2 (1D), 3 (2D),$ or 4(3*D*) and in an unstructured mesh $C_{d}=2$ in 1D but $C_{d}$ depends on the particular mesh in 2D and 3D. The mean value vectors $\mathbf {u}$ and $\tilde{\mathbf {u}}$ have the dimension *MN*.

In order to estimate the computational work in the steps of the algorithm, we assume that $\nu _i$ depends on a limited number of $u_{jk}$ independent of *M*. Then, there are also a limited number of derivatives $\nu _{i,j}$ different from zero and independent of *M*. Thus, the work to compute the right-hand side (RHS) in step 1 in (60) is independent of *M* and *N* and it is computed once for every species *i* and voxel *k*, i.e., *MN* times. Since there are a limited number of nonzeros in $\nu _{i,\alpha }$, the sums and $\mathbf W$ in step 2 in (61) are computed independently of *M* and *N*. Hence, the work is proportional to $M^2N$ for the covariances between the species in every voxel. In step 3 in (62), $M^2$ covariances are computed for every combination of voxels *k* and *l* where $\tilde{\Xi }$ is nonzero. This is the case when *k* and *l* are neighbors and each *k* has a limited number of neighbors. This number is independent of *N*. Therefore, the work to compute the full RHS in step 3 is of ${\mathcal O}(M^2N)$. The number of $D_{k\beta }\ne 0$ in the RHS in step 4 in (63) is independent of *N* according to the previous paragraph. The work to determine all derivatives of $u_{ik}$ is then proportional to *MN*. For $\Xi _{ii;kl}$ to be nonzero in step 5 in (64), voxels *k* and *l* are neighbors, $l\in \mathcal {J}(k)$. Furthermore, the products in the sums are nonzero only if $\beta \in \mathcal {J}(k)\cap \mathcal {J}(l)$. The work to calculate the sums is independent of *N*, and the RHS is computed ${\mathcal O}(MN)$ times. In the same manner, the RHS in step 6 in (65) is computed ${\mathcal O}(M^2N)$ times. The conclusion is that the work to determine the RHS in the ODEs for $u_{ik}$ and $\hat{\Xi }_{ij;kl}$ in the algorithm has linear complexity in *N* and is proportional to $M^2N$.

Since there are additional administrative costs in Algorithm 1, the straightforward algorithm ignoring the sparsity of $\Xi$ will be faster when $N<N_*$ for some small $N_*$ which is problem dependent. However, for $N>N_*$ Algorithm 1 will be the winner and its advantage is greater, the greater the *N* is.

If the diffusion coefficient is different for different species *i*, then $D_{k\beta }$ and $D_{l\beta }$ in steps 4, 5 and 6 would depend on *i* but the algorithm and its properties remain the same.

In summary, the algorithm in words is for one timestep $\Delta t$ from $t^n$ to $t^{n+1}=t^n+\Delta t$:

1. Solve the ODE in (60) numerically for the mean values with initial data $\mathbf {u}(t^n)$ to obtain $\tilde{\mathbf {u}}(t)$
2. Solve the ODE in (61) numerically for the covariances between the species in the same voxel *k* with $\tilde{\mathbf {u}}$ from step 1 and initial data $\varvec{\Xi }(t^n)$ to determine $\tilde{\varvec{\Xi }}_{\cdot \cdot ;kk}(t)$
3. Solve the ODE in (62) numerically for the covariances between the species in different voxels *k* and *l* satisfying $l\in \mathcal {J}(k)$ with $\tilde{\mathbf {u}}$ from step 1 and initial data $\varvec{\Xi }(t^n)$ to determine $\tilde{\varvec{\Xi }}_{\cdot \cdot ;kl}(t)$
4. Solve the ODE in (63) numerically for the mean values with initial data $\tilde{\mathbf {u}}(t^{n+1})$ from (60) to obtain ${\mathbf {u}}(t)$ and ${\mathbf {u}}(t^{n+1})$
5. Solve the ODE in (64) numerically for the covariances between voxels *k* and *l* satisfying $l\in \mathcal {J}(k)$ for the same species *i* with ${\mathbf {u}}$ from step 4 and initial data $\tilde{\varvec{\Xi }}(t^{n+1})$ from steps 2 and 3 to determine $\varvec{\Xi }_{ii;\cdot \cdot }(t^{n+1})$
6. Solve the ODE in (65) numerically for the covariances between voxels *k* and *l* satisfying $l\in \mathcal {J}(k)$ for different species *i* and *j* with ${\mathbf {u}}$ from step 4 and initial data $\tilde{\varvec{\Xi }}(t^{n+1})$ from steps 2 and 3 to determine ${\varvec{\Xi }}_{ij;\cdot \cdot }(t^{n+1})$

In the first three steps, the mean values and the covariances change due to the reactions and in the last three steps due to the diffusion.

Theorem 1 and numerical experiments in Sect. 6.1 indicate that the accuracy in $\hat{\Xi }$ increases when the dimension grows. By storing and updating only the sparse approximation in steps 3, 5 and 6 in Algorithm 1, considerable savings are possible in computing time and computer memory when *N* is large, e.g., in 3D.

### Example

Consider the reversible reaction for association and dissociation of the species *A*, *B*,  and *C*

66 $$\begin{aligned} A+B \overset{k_a'}{\underset{k_d'}{\rightleftharpoons }} C, \end{aligned}$$

with copy numbers $\varvec{\mu }_{\cdot j}^T=(a_j, b_j, c_j)$ in voxel *j* and propensities and state change vectors

67 $$\begin{aligned} v_1=k_a'a_jb_j,\quad \mathbf n_1^T=(-1, -1, 1),\quad v_2=k_d'c_j,\quad \mathbf n_2^T=(1, 1, -1). \end{aligned}$$

The macroscopic reaction coefficients are $k_a=V_kk_a'$ and $k_d=k_d'$. Then, Eq. (21) for the concentrations in step 1 of the above algorithm in $\mathcal {V}_k$ is

68 $$\begin{aligned} \dot{\mathbf {u}}_{\cdot k}=\left( \begin{array}{c} -k_au_{1k}u_{2k}+k_du_{3k}\\ -k_au_{1k}u_{2k}+k_du_{3k}\\ k_au_{1k}u_{2k}-k_du_{3k} \end{array}\right) ,\quad k=1,\ldots ,N. \end{aligned}$$

Order the means and the covariances such that

69 $$\begin{aligned} \mathbf {u}=\left( \begin{array}{c} \mathbf {u}_{1 \cdot }\\ \mathbf {u}_{2 \cdot }\\ \mathbf {u}_{3 \cdot } \end{array}\right) ,\quad \varvec{\Xi }=\left( \begin{array}{ccc} \varvec{\Xi }_{11;\cdot \cdot }&{}\varvec{\Xi }_{12;\cdot \cdot }&{}\varvec{\Xi }_{13;\cdot \cdot }\\ \varvec{\Xi }_{21;\cdot \cdot }&{}\varvec{\Xi }_{22;\cdot \cdot }&{}\varvec{\Xi }_{23;\cdot \cdot }\\ \varvec{\Xi }_{31;\cdot \cdot }&{}\varvec{\Xi }_{32;\cdot \cdot }&{}\varvec{\Xi }_{33;\cdot \cdot } \end{array}\right) . \end{aligned}$$

The Jacobian $\mathbf {J}$ of the propensities with $J_{ij}=\nu _{i,j}$ in (31) and the source term in step 2 in $\mathcal {V}_k$ are

70 $$\begin{aligned} \mathbf {J}= & {} \left( \begin{array}{ccc} -k_au_{2k}&{}-k_au_{1k}&{}k_d\\ -k_au_{2k}&{}-k_au_{1k}&{}k_d\\ k_au_{2k}&{}k_au_{1k}&{}-k_d \end{array}\right) ,\nonumber \\ g_k(\mathbf {u})= & {} V_k^{-1}(k_au_{1k}u_{2k}+k_du_{3k}),\quad V_k^{-2}\mathbf W=g_k(\mathbf {u})\mathbf n_1\mathbf n_1^T, \end{aligned}$$

since $\mathbf n_1\mathbf n_1^T=\mathbf n_2\mathbf n_2^T$. Introduce $\mathbf {K}$ and $\mathbf {G}$ using the identity matrix $\mathbf I_N$ of size *N*, $\mathbf {J}$, and $\mathbf W$ in (70)

71 $$\begin{aligned} \mathbf {K}= & {} \left( \begin{array}{ccc} -k_a\mathrm{diag}(\mathbf {u}_{2 \cdot })&{}-k_a\mathrm{diag}(\mathbf {u}_{1 \cdot })&{}k_d\mathbf I_N\\ -k_a\mathrm{diag}(\mathbf {u}_{2 \cdot })&{}-k_a\mathrm{diag}(\mathbf {u}_{1 \cdot })&{}k_d\mathbf I_N\\ k_a\mathrm{diag}(\mathbf {u}_{2 \cdot })&{}k_a\mathrm{diag}(\mathbf {u}_{1 \cdot })&{}-k_d\mathbf I_N \end{array}\right) \nonumber \\= & {} \left( \begin{array}{c} -1\\ -1\\ 1 \end{array}\right) \otimes (k_a\mathrm{diag}(\mathbf {u}_{2 \cdot })k_a\mathrm{diag}(\mathbf {u}_{1 \cdot })-k_d\mathbf I_N),\nonumber \\ \mathbf {G}(\mathbf {u})= & {} \mathbf n_1\mathbf n_1^T\otimes \mathrm{diag}(\mathbf g(\mathbf {u})), \end{aligned}$$

where $\otimes$ denotes the Kronecker product. Then, the equation in steps 2 and 3 in matrix form is

72 $$\begin{aligned} \dot{\tilde{\varvec{\Xi }}}=\mathbf {K}\tilde{\varvec{\Xi }}+(\mathbf {K}\tilde{\varvec{\Xi }})^T+\mathbf {G}(\tilde{\mathbf {u}}). \end{aligned}$$

Define the matrices $\mathbf {D}_3$ and $\mathbf {H}$ by

73 $$\begin{aligned} \mathbf {D}_3= & {} \left( \begin{array}{ccc} \mathbf {D}&{}\mathbf {0}&{}\mathbf {0}\\ \mathbf {0}&{}\mathbf {D}&{}\mathbf {0}\\ \mathbf {0}&{}\mathbf {0}&{}\mathbf {D}\end{array}\right) =\mathbf I_3\otimes \mathbf {D},\nonumber \\ \mathbf {H}(\mathbf {u})= & {} \left( \begin{array}{ccc} \mathbf {H}_{1;\cdot \cdot }&{}\mathbf {0}&{}\mathbf {0}\\ \mathbf {0}&{}\mathbf {H}_{2;\cdot \cdot }&{}\mathbf {0}\\ \mathbf {0}&{}\mathbf {0}&{}\mathbf {H}_{3;\cdot \cdot } \end{array}\right) ,\quad H_{i;kl}=D_{kl}V_l^{-1}(u_{ik}+u_{il}). \end{aligned}$$

The submatrix $\mathbf {D}$ is the approximation of the Laplacian in (31) and (39). If the diffusion varies between the species, then $\mathbf {D}$ in (73) would be replaced by $\gamma _i/\gamma \mathbf {D}, i=1,2,3,$ on the diagonal. The sparsity or nonzero pattern in $\mathbf {H}_{i;\cdot \cdot }$ is the same as in $\mathbf {D}$. The diffusion equation for the mean values in step 4 in Algorithm 1 is as in (31)

74 $$\begin{aligned} \dot{\mathbf {u}}=\mathbf {D}_3\mathbf {u}. \end{aligned}$$

The matrix form of steps 5 and 6 in Algorithm 1 is (cf. (38) and (39))

75 $$\begin{aligned} \dot{\varvec{\Xi }}=\mathbf {D}_3\varvec{\Xi }+(\mathbf {D}_3\varvec{\Xi })^T-\mathbf {H}(\mathbf {u}). \end{aligned}$$

In 1D, $\mathbf {D}$ is a tridiagonal matrix and if $V=\Delta x$ is constant then

$$\begin{aligned} D_{\alpha k}= & {} D_{k\alpha }=\gamma /\Delta x^2,\quad \alpha =k+1,\quad k=1,\ldots ,N-1,\nonumber \\ D_{k k}= & {} -2\gamma /\Delta x^2,\quad k=2,\ldots ,N-1,\quad D_{1 1}=D_{N N}=-\gamma /\Delta x^2. \end{aligned}$$

## Numerical Results

The algorithm is tested for computing the mean and the approximation of the covariance in the LNA of systems with diffusion in 1D, 2D, and 3D and a system in 2D with the reversible reaction (66).

### Diffusion

A Cartesian grid in *d* dimensions is generated with a constant step size $\Delta x$ and a diffusion coefficient $\gamma =0.01$. The number of dimensions is $d=1,2,3,$ and the domain is the unit cube $[0, 1]^d$. The number of grid points is $n=1/\Delta x+1$ in each dimension yielding $N=n^d$ components in $\mathbf {u}$. A straightforward implementation of Algorithm 1 in steps 5 and 6 will generate $N^2$ elements in $\varvec{\Xi }$. By updating only those elements of $\varvec{\Xi }$ that correspond to nonzeros in $\mathbf {D}$ and $\mathbf {S}$, the number of nonzero elements in the approximation $\hat{\varvec{\Xi }}$ will be of ${\mathcal O}(N)$.

The initial data $\mathbf {u}(0)$ are sampled from a uniform distribution $u_k(0)\sim \mathcal {U}[0, 1]$ and $\varvec{\Xi }=\mathbf {0}$. The ODEs in (74) and (75) are solved numerically for $t\ge 0$ by the forward Euler method for simplicity. Then, the RHS in each step of Algorithm 1 is evaluated once requiring a computational work proportional to $M^2N$ in every timestep from $t^n$ to $t^{n+1}$. Better numerical accuracy is achieved by splitting the computations according to Strang (1968) as in (11) and by using a higher-order method. Better numerical stability is obtained by an implicit method.

#### 1D

**Fig. 2:**
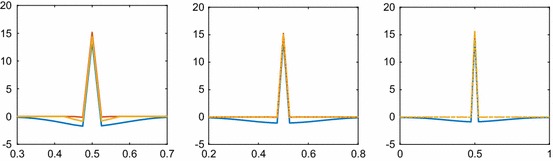
Comparison of the covariance $\Xi (0.5, x_2, t)$ for diffusion in 1D with $x_2\in [0, 1]$ on the abscissa at different time points and different approximations: without sparse approximation (blue), with two extra diagonals in $\hat{\Xi }(0.5,x_2)$ (red), and with four extra diagonals in $\check{\Xi }(0.5,x_2)$ (yellow). The time is $t=8$ (left), $t=20$ (middle), and $t=40$ (right)

The covariance $\Xi (x_1, x_2, t)$ is computed in 1D on a grid with $\Delta x=0.025$ and $N=n=41$ using the full $\varvec{\Xi }$ without zeros, the sparse $\hat{\varvec{\Xi }}$ with the same nonzero pattern as $\mathbf {S}$, i.e., the diagonal, the subdiagonal, and the superdiagonal are nonzero in a tridiagonal matrix as proposed in Sect. 6.1, and the sparse $\check{\varvec{\Xi }}$ where another two diagonals below and above the diagonal are nonzero in a pentadiagonal matrix. One row of the three matrices is shown in Fig. 2. The approximations $\hat{\Xi }(0.5, x_2, t)$ and $\check{\Xi }(0.5, x_2, t)$ agree fairly well with $\Xi (0.5, x_2, t)$ in particular for larger *t* in Fig. 2.

The PDF of the multivariate normal distribution $\mathcal {N}(\mathbf {u}, \varvec{\Xi })$ is

76 $$\begin{aligned} p(\varvec{\eta }, t)=\frac{1}{(2\pi )^{N/2}\sqrt{\det \varvec{\Xi }}}\exp \left( -\frac{1}{2}(\varvec{\eta }-\mathbf {u})^T\varvec{\Xi }^{-1}(\varvec{\eta }-\mathbf {u})\right) . \end{aligned}$$

The covariance matrix is factorized by $\varvec{\Xi }=\mathbf {Q}\varvec{\Lambda }\mathbf {Q}^T$ where $\mathbf {Q}$ is orthogonal and $\varvec{\Lambda }$ has the positive eigenvalues of $\varvec{\Xi }$ on the diagonal. The expression in (76) in the exponential defines surfaces of ellipsoids in $\mathbb R^N$ with equal probability, and the eigenvalues of $\varvec{\Xi }$ are the lengths of the principal axes of the ellipsoids. Another way of comparing $\varvec{\Xi }$ and its approximations is then to compare the eigenvalues to see the difference between the lengths of these axes.

**Fig. 3:**
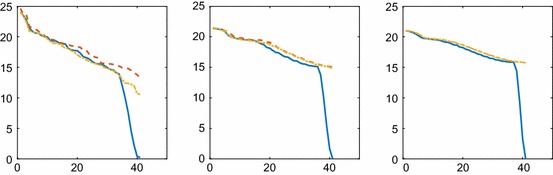
Comparison of the *N* eigenvalues of the covariance $\Xi (0.5, x_2, t)$ for diffusion in 1D at different time points and different approximations: without sparse approximation (solid blue), with two extra diagonals in $\hat{\Xi }$ (dashed red), and with four extra diagonals in $\check{\Xi }$ (dash-dotted yellow). The time is $t=8$ (left), $t=20$ (middle), and $t=40$ (right). The curves for $\hat{\varvec{\Xi }}$ and $\check{\varvec{\Xi }}$ at $t=40$ are indistinguishable with the precision in the figure

The eigenvalues of $\varvec{\Xi }, \hat{\varvec{\Xi }},$ and $\check{\varvec{\Xi }}$ are displayed in Fig. 3. They also agree well except for the one or two smallest ones in the figure. Using five diagonals and four neighbors in $\check{\varvec{\Xi }}$ improves the approximation somewhat compared to $\hat{\varvec{\Xi }}$. Including more than the nearest neighbors in 2D and 3D with an unstructured mesh is possible but makes the algorithm more complicated.

#### 2D and 3D

**Fig. 4:**
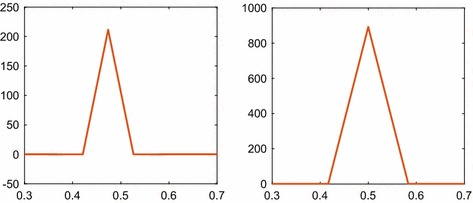
The covariances $\Xi (\mathbf {x}_1, \mathbf {x}_2, t)$ for diffusion computed with the full matrix $\varvec{\Xi }$ and its sparse approximation $\hat{\varvec{\Xi }}$ in 2D (left) and 3D (right). The coordinate $\mathbf {x}_1$ chosen in the middle of the domain and with one degree of freedom in $\mathbf {x}_2$ on the abscissa at $t=2.5$ with $\Delta x_2=1/20$ (left) and $t=0.2$ with $\Delta x_2=1/12$ (right). The difference between $\varvec{\Xi }$ and $\hat{\varvec{\Xi }}$ is not discernible in the figures

In 2D, $\Xi (\mathbf {x}_1, \mathbf {x}_2, t)$ is computed with the full $\varvec{\Xi }$ matrix and with the approximation $\hat{\varvec{\Xi }}$ that has the same sparsity pattern as $\mathbf {S}$ on a grid with $\Delta x=0.05$ and $N=n^2=441$. One row of $\varvec{\Xi }$ corresponds to one coordinate $\mathbf {x}_{1k}$ and its covariance with the 2D $\mathbf {x}_2$. The variance is high at $\Xi (\mathbf {x}_1, \mathbf {x}_1, t)$, and the covariance $\Xi (\mathbf {x}_1, \mathbf {x}_2, t)$ is very low when $\mathbf {x}_1\ne \mathbf {x}_2$. This is depicted in the left panel of Fig. 4 where $\mathbf {x}_1$ and $x_{22}$ are fixed and $x_{21}$ varies in $\mathbf {x}_2^T=(x_{21}, x_{22})$. The differences in covariance between $\varvec{\Xi }$ and $\hat{\varvec{\Xi }}$ are very small and not visible in the figure. Since $\varvec{\Xi }$ is symmetric, the result is similar in other directions in $\mathbf {x}_2$ and for other $\mathbf {x}_1$. The steady-state solution (40) with $u=0.5$ and $V=1/400$ is here 200.

One section of the 3D covariances $\varvec{\Xi }$ and $\hat{\varvec{\Xi }}$ is shown in the right panel of Fig. 4. As in 2D, $\mathbf {x}_1$, $x_{22}$, and $x_{23}$ in $\mathbf {x}_2^T=(x_{21}, x_{22}, x_{23})$ are fixed and $\Xi (\mathbf {x}_1, \mathbf {x}_2, t)$ is plotted as a function of $x_{21}$. The step size in the grid is $\Delta x=0.0833$ and $N=n^3=2197$. After a short time, the covariances of $\varvec{\Xi }$ and $\hat{\varvec{\Xi }}$ agree very well as in 2D. The stationary solution in (40) with the data here is 864.

The scaled difference $\Delta \varvec{\Xi }$ between the covariances $\varvec{\Xi }$ and $\hat{\varvec{\Xi }}$ is defined by

77 $$\begin{aligned} \varvec{\Xi }=\hat{\varvec{\Xi }}(\mathbf I_N+\Delta \varvec{\Xi }),\quad \Delta \varvec{\Xi }=\hat{\varvec{\Xi }}^{-1}(\varvec{\Xi }-\hat{\varvec{\Xi }}). \end{aligned}$$

**Fig. 5:**
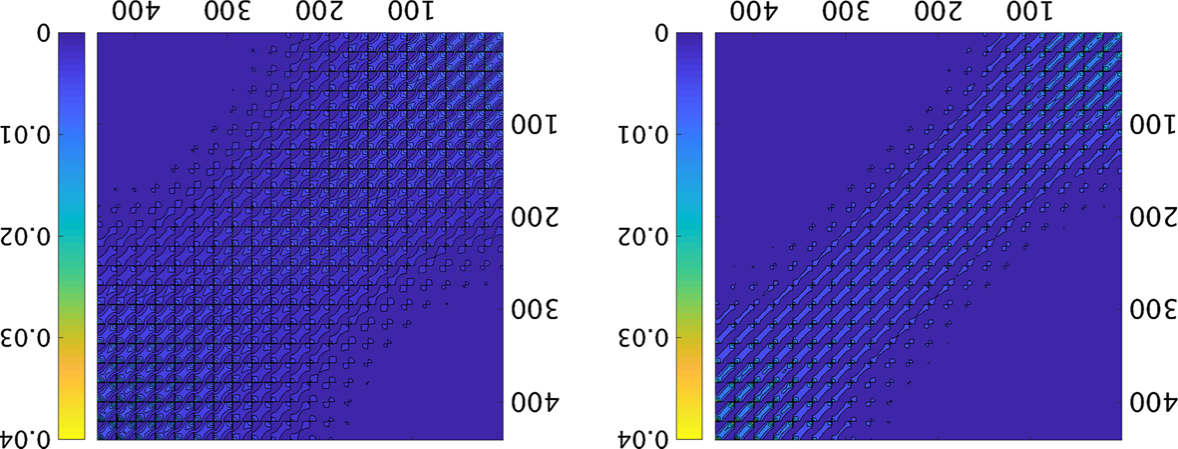
Scaled difference $\Delta \Xi _{ij}$ of the covariance matrices $\varvec{\Xi }$ and $\hat{\varvec{\Xi }}$ in (77) when $i,j=1,\ldots ,N$ for diffusion in 2D at $t=2.5$ (left) and $t=5$ (right)

The dominant elements in $\hat{\varvec{\Xi }}$ are the variances on the diagonal. With small elements in $\Delta \varvec{\Xi }$ compared to 1, the difference between the covariances in $\varvec{\Xi }$ and $\hat{\varvec{\Xi }}$ is small relative to the variances in $\hat{\varvec{\Xi }}$. In Fig. 5, $\Delta \varvec{\Xi }$ for the 2D example is shown at two time points. The values of $\Delta \varvec{\Xi }$ are low in blue color in most parts of the matrix. The peaks in the left panel are at 0.035 in isolated points. In the right panel, $\max \Delta \Xi _{ij}<0.02$.

**Fig. 6:**
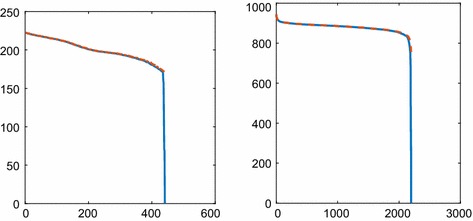
Comparison of the *N* eigenvalues of the covariance matrices $\varvec{\Xi }$ and $\hat{\varvec{\Xi }}$ for diffusion in 2D at $t=2.5$ (left) and 3D at $t=0.2$ (right) and different approximations: with sparse approximation (dashed red) and without (solid blue)

The eigenvalues of $\varvec{\Xi }$ and $\hat{\varvec{\Xi }}$ in 2D and 3D are compared in Fig. 6. The sparse approximation captures all the eigenvalues except for one or two of the smallest ones.

**Fig. 7:**
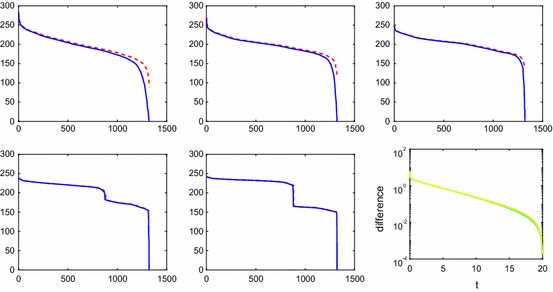
Comparison of the *MN* eigenvalues of the covariance matrices $\varvec{\Xi }$ and $\hat{\varvec{\Xi }}$ for the reaction (66) and diffusion in 2D at $t=0.25$ (upper left), $t=0.5$ (upper middle), $t=1$ (upper right), $t=3$ (lower left), $t=6$ (lower middle) and different approximations: with sparse approximation (solid blue) and without (dashed red). The convergence of the solution to the steady state for the species *A*, *B*,  and *C* as a function of *t* (lower right)

The covariance of the fluctuations in concentration between different parts of the domain is well approximated by the sparse $\hat{\varvec{\Xi }}$, especially in 2D and 3D in Figs. 4 and 6. This is expected from Theorem 1 in Sect. 4 where the decay of $\Xi (\mathbf {x}_1, \mathbf {x}_2, t)$ is slower in 1D than in 2D and 3D when $\varvec{\xi }_1=\frac{1}{\sqrt{2}}(\mathbf {x}_1-\mathbf {x}_2)$ is growing.

### Reactions and Diffusion in 2D

The time evolution of the chemical reaction (66) on the Cartesian mesh in 2D in Sect. 6.1.2 is computed with the LNA as in the example in Sect. 5.1. The parameters are $k_a=k_d=0.1$, and the diffusion is low with $\gamma =0.01$. The dimension of $\mathbf {u}$ is $MN=Mn^2=1323$, and the initial values in $\mathbf {u}(0)$ are uniformly distributed between 0 and 1 and $\varvec{\Xi }(0)=\mathbf {0}$.

The eigenvalues of the covariances with the full matrix $\varvec{\Xi }$ and with the sparse matrix $\hat{\varvec{\Xi }}$ are compared at different *t* in Fig. 7. An approximate stationary solution $\mathbf {u}_\infty$ is determined at $t=20$. The convergence of the three subvectors $\mathbf {u}_{1 \cdot }, \mathbf {u}_{2 \cdot },$ and $\mathbf {u}_{3 \cdot }$ in (69) corresponding to the concentrations of *A*, *B*,  and *C* is displayed in the lower right panel in the figure. The difference between $\mathbf {u}$ and $\mathbf {u}_\infty$ is measured in $\Vert \cdot \Vert$ for the species. In the resolution of the figure, it is not possible to distinguish between the differences in convergence between the species. The balance equation $k_a \bar{a} \bar{b}=k_d \bar{c}$ is satisfied with a relative error less than 0.008 by the mean values $\bar{a}, \bar{b},$ and $\bar{c}$ of the components in $\mathbf {u}_{i \cdot }(20), \, i=1,2,3$. At $\infty$, $\mathbf {u}_{\infty 1 \cdot }, \mathbf {u}_{\infty 2 \cdot },$ and $\mathbf {u}_{\infty 3 \cdot }$ are constant in space.

The convergence plot in Fig. 7 shows that the variation in the solution is larger for small *t* and decreases with *t*. The covariances $\varvec{\Xi }$ and $\hat{\varvec{\Xi }}$ agree well for large eigenvalues for small *t* and they agree well for all eigenvalues when *t* grows. The off-diagonal submatrices $\varvec{\Xi }_{ij;\cdot \cdot },\; i\ne j,$ in (69) are comparable in size to the diagonal submatrices $\varvec{\Xi }_{ii;\cdot \cdot }$ when *t* is small but as *t* grows $\varvec{\Xi }_{ii;\cdot \cdot },\; i=1,2,3,$ will dominate and be closer and closer to diagonal matrices. There is a jump in the spectrum for larger *t*, e.g., at $t=6$. This is explained by the difference in the size of the stationary values $\bar{a}, \bar{b},$ and $\bar{c}$ where $\bar{a}\approx \bar{b},$ and $\bar{c}/\bar{a}\approx 0.6$. The approximation in the covariance equation behaves in the same way with reactions as in Sect. 6.1.2 without the reactions.

## Conclusions

The master equation is a model for biochemical reactions and diffusion but the numerical solution of it is impossible except for simple, well-stirred systems with special properties. An alternative for large systems with spatial variation is to use the linear noise approximation (LNA). We have derived the equations for the LNA for diffusion and chemical reactions on general meshes. The reactions involve *M* species, and the mesh consists of *N* voxels. The covariance of the concentrations is approximated by a sparse representation in an algorithm such that the computational complexity is reduced from ${\mathcal O}(M^2 N^2)$ in a straightforward implementation to ${\mathcal O}(M^2 N)$ here. Also the memory to store the solution is reduced in the same way. The approximation is supported by analytical expressions showing that the higher the dimension is, the better the approximation is. Consequently, the quality of the approximation and the savings in work and storage are more prominent in 3D when *N* is large. The accuracy of the approximation is evaluated by comparing the elements and the eigenvalues of the full covariance matrix and its sparse approximation in numerical examples with only diffusion in 1D, 2D, and 3D and an example in 2D with a reversible reaction and slow diffusion.
